# Selective autophagic receptor p62 regulates the abundance of transcriptional coregulator ARIP4 during nutrient starvation

**DOI:** 10.1038/srep14498

**Published:** 2015-09-28

**Authors:** Megumi Tsuchiya, Shin Isogai, Hiroaki Taniguchi, Hidehito Tochio, Masahiro Shirakawa, Ken-ichirou Morohashi, Yasushi Hiraoka, Tokuko Haraguchi, Hidesato Ogawa

**Affiliations:** 1 Graduate School of Frontier Biosciences, Osaka University, 1-3 Yamadaoka, Suita 565-0871, Japan; 2 Graduate School of Engineering, Kyoto University, Kyoto 615-8510, Japan; 3 Laboratory for Genetic Code, Graduate School of Life and Medical Sciences, Doshisha University, 1-3 Tatara Miyakodani, Kyotanabe 610-0394, Japan; 4 Department of Molecular Biology, Graduate School of Medical Sciences, Kyushu University, Fukuoka 812-8582, Japan; 5 Advanced ICT Research Institute Kobe, National Institute of Information and Communications Technology, Kobe 651-2492, Japan

## Abstract

Transcriptional coregulators contribute to several processes involving nuclear receptor transcriptional regulation. The transcriptional coregulator androgen receptor-interacting protein 4 (ARIP4) interacts with nuclear receptors and regulates their transcriptional activity. In this study, we identified p62 as a major interacting protein partner for ARIP4 in the nucleus. Nuclear magnetic resonance analysis demonstrated that ARIP4 interacts directly with the ubiquitin-associated (UBA) domain of p62. ARIP4 and ubiquitin both bind to similar amino acid residues within UBA domains; therefore, these proteins may possess a similar surface structure at their UBA-binding interfaces. We also found that p62 is required for the regulation of ARIP4 protein levels under nutrient starvation conditions. We propose that p62 is a novel binding partner for ARIP4, and that its binding regulates the cellular protein level of ARIP4 under conditions of metabolic stress.

Transcriptional coregulators are required for the efficient transcriptional regulation of nuclear receptors. Coregulators play various important roles in transcriptional regulation, including direct interactions with basal transcription factors, covalent modification of histones and other proteins, and modification of chromatin conformation (through chromatin remodelling)^1^,^2^,^3^,^4^. Chromatin remodelling is the dynamic modification of chromatin architecture that controls access to genomic DNA by the transcription machinery proteins, thereby controlling gene expression^5^,^6^,^7^,^8^.

Androgen receptor-interacting protein 4 (ARIP4) was identified as a transcriptional coregulator and is classified as part of the Rad54 subfamily of the SNF2-type chromatin remodelling factor superfamily^9^,^10^. This subfamily includes Rad54 and ATRX (α-thalassemia/mental retardation syndrome X-linked)^11^,^12^. Rad54 interacts with Rad51 and this interaction enhances the ATPase activity of Rad54 and the ability of Rad51 to form cruciform and D-loop DNA conformations^13^,^14^,^15^. Moreover, biochemical studies identified DAXX (death domain-associated protein) as an interacting partner for ATRX. ATRX complexes containing DAXX catalyse the deposition and remodelling of H3.3-containing nucleosomes^16^,^17^,^18^. Similarly, it is likely that ARIP4 also possesses a stable interacting partner important for ARIP4 regulation.

ARIP4 was shown to interact with sumoylated nuclear receptor adrenal 4 binding protein/steroidogenic factor 1(Ad4BP/SF-1), liver receptor homolog-1 (LRH-1), glucocorticoid receptor (GR) and androgen receptor (AR)^10^. ARIP4 belongs to the SNF2 family and possesses double-stranded DNA-dependent ATPase activity. The ATPase activity of ARIP4 is stimulated in the presence of both sumoylated Ad4BP/SF-1 and double-stranded DNA containing the Ad4BP/SF-1 binding site^10^. Similar to several other ATP-dependent chromatin remodelling proteins, ARIP4 generates superhelical torsion within linear DNA fragments in an ATP-dependent manner resulting in cruciform formation^9^. As a transcriptional coregulator, ARIP4 acts as a repressor that modulates SUMO-mediated fine-tuning of Ad4BP/SF-1 target gene transactivation^10^.

Regarding the physiological functions of ARIP4, Arip4^−/−^ mouse embryos are embryonic lethal by embryonic day 11.5 (E11.5). Mouse embryonic fibroblasts (MEFs) isolated from Arip4-null mouse embryos exhibited increased apoptosis and decreased DNA synthesis compared with wild-type MEFs, indicating that ARIP4 plays a central role in cell survival and cell homeostasis^19^. Despite this *in vitro* and *in vivo* evidence, little is known about ARIP4 response mechanisms to cellular signals. To address this question, it is important to identify and analyse the proteins that interact with and transduce cellular signals to ARIP4. In this regard, previous studies have identified several proteins that interact with ARIP4, such as SUMO2, ATNX1 and Dyrk1A^20^,^21^,^22^. Dyrk1A acts as a coregulator for nuclear receptors such as AR and GR. Furthermore, Dyrk1A and ARIP4 have a synergistic effect on AR and GR transactivation^22^. It is interesting to note that ARIP4 acts as a coactivator or corepressor on target promoters; the function it carries out may be selected in a context-dependent manner^10^. Given that various interacting factors may regulate ARIP4 function, their analysis will in turn contribute to the understanding of ARIP4 regulatory mechanisms.

In the present study, we biochemically purified ARIP4 protein complexes. One of the components of this protein complex was identified as p62 (also known as SQSTM1, A170, or ZIP); p62 is a well-known autophagic receptor that binds ubiquitinated proteins in the early process of autophagy^23^,^24^,^25^. Our biochemical and nuclear magnetic resonance (NMR) studies of the interaction between ARIP4 and p62 showed that ARIP4 binds directly to p62 through a novel interaction domain. This domain possesses a similar binding profile to that of ubiquitin for the ubiquitin-associated (UBA) domain of p62. Given that the role of autophagy in starvation response is well established^26^,^27^,^28^, we also examined whether ARIP4 governs p62 function under starvation conditions.

## Results

### Identinfication of ARIP4-binding proteins

**Figure 1:**
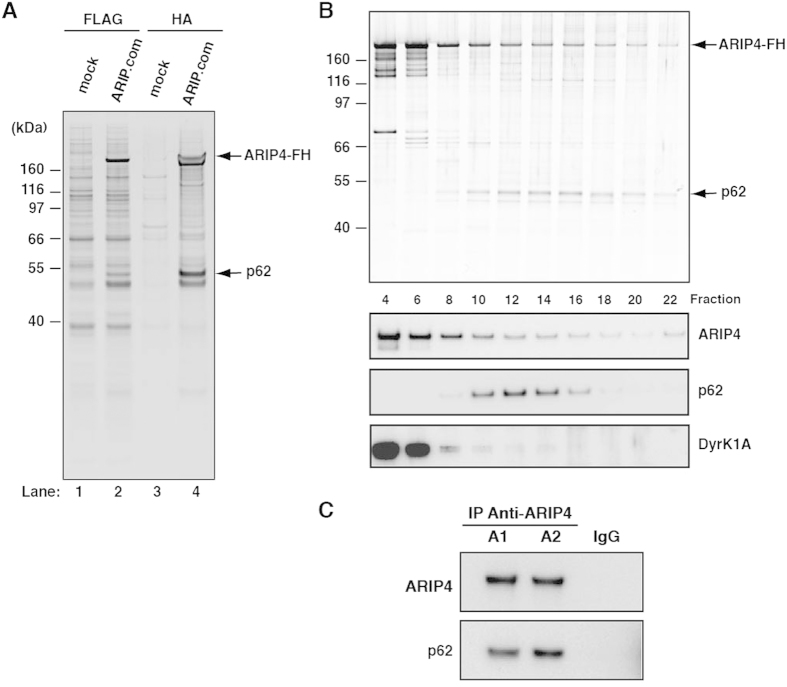
Purification of ARIP4-containing complexes. (**A**) FLAG-HA epitope-tagged ARIP4 (ARIP4-FH) was expressed and purified from HeLa cells by immunoprecipitation with an antibody specific to FLAG (lane 2), followed by an antibody specific to HA (lane 4). The purified proteins were resolved by NuPAGE gel electrophoresis and visualised by silver staining. As a control, a mock purification was performed on HeLa cells not expressing exogenous proteins (lanes 1 and 3). The ~54-kDa protein that directly associated with ARIP4-FH (indicated with an arrow), was identified as p62 by mass spectrometry analysis. Molecular weight standards are shown on the left. (**B**) FLAG-purified proteins were separated with a 10–40% glycerol gradient by centrifugation. The proteins in each fraction were purified with anti-HA antibody and the fractions (top to bottom) were resolved by NuPAGE gel electrophoresis and visualised by silver staining (upper panel) and immunoblotted with ARIP4-, p62- and Dyrk1A-specific antibodies (lower panels). (**C**) Interaction between endogenous p62 and ARIP4. HeLa nuclear extracts were immunoprecipitated with anti-ARIP4 antibody (A1 and A2 antibodies were produced by different rabbits) or rabbit IgG as a control. The immunoprecipitates were analysed by western blotting with the indicated antibodies.

ARIP4-containing complexes were purified from nuclear extracts of HeLa cells stably expressing FLAG-haemagglutinin (HA) epitope-tagged ARIP4 (ARIP4-FH) by FLAG immunoprecipitation followed by HA immunoprecipitation. The proteins in these isolated complexes are shown in Fig. 1A, lanes 2 and 4 (ARIP.com). Mock purification (cells not expressing exogenous proteins) did not isolate these proteins (Fig. 1A, lanes 1 and 3), suggesting that the isolated proteins are specific to ARIP4. We further analysed the isolated proteins using matrix-assisted laser desorption ionization time-of-flight mass spectrometry (MALDI-TOF MS) and identified the 54-kDa protein (Fig. 1A, lanes 2 and 4) as p62^23^,^24^,^25^. We also identified an 86-kDa protein as Dyrk1A^22^, and a 40-kDa protein as HAN11 (also known as WDR68)^29^,^30^,^31^.

Next, we analysed the potential interactions between p62 and ARIP4 in HeLa cells. To further separate these proteins, the anti-FLAG purified fractions were applied to glycerol gradient centrifugation. The proteins contained in each fraction were immunoprecipitated using an antibody against HA. The resulting precipitates were visualised with silver staining (Fig. 1B, top) and western blotting (Fig. 1B, bottom). Western blot analysis identified p62 as a single peak in the higher molecular weight fractions (fractions 10–16; Fig. 1B), whereas ARIP4-FH was observed in both lower and higher molecular weight fractions (fractions 4–8 and fractions 10–16; Fig. 1B). Conversely, the known ARIP4-interacting protein, Dyrk1A, was detected in low molecular weight fractions only (fractions 4–6). These data suggest that, under these conditions, binding of p62 to ARIP4 is more stable than that of Dyrk1A to ARIP4. To further confirm the interaction between ARIP4 and p62 under physiological conditions, we examined whether endogenous ARIP4 could bind endogenous p62. We performed immunoprecipitation experiments using an anti-ARIP4 antibody and nuclear extracts of HeLa cells not expressing exogenous proteins, and found that p62 co-immunoprecipitated with ARIP4 (Fig. 1C). These findings suggest that endogenous ARIP4 binds to p62.

### Colocalisation of p62 with ARIP4 in U2OS cells

**Figure 2:**
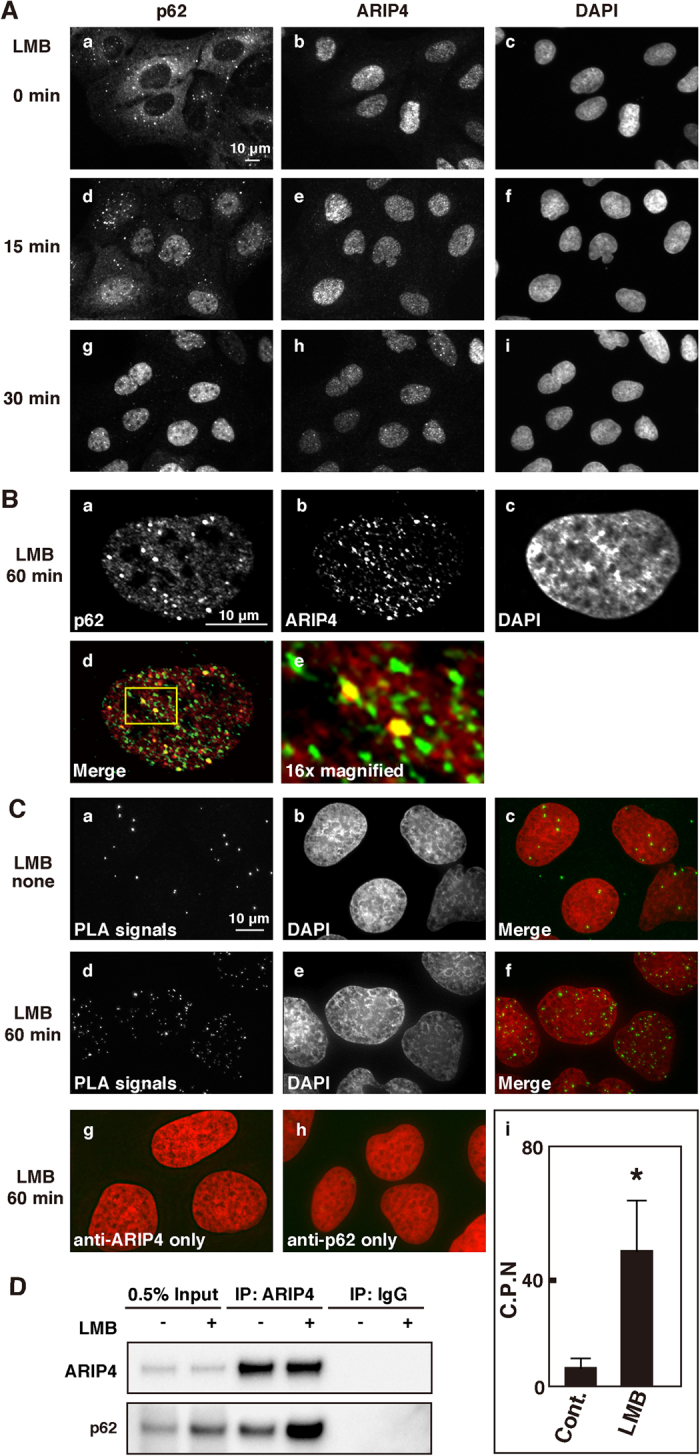
LMB induces the nuclear localisation of p62 in U2OS cells. (**A**) U2OS cells were treated with LMB (100 ng/mL), fixed at the indicated time points, stained with anti-p62 (a, d and g) and anti-ARIP4 antibodies (b, e and h), and visualised using Alexa488-conjugated secondary antibody and Cy3-conjugated secondary antibody, respectively. Nuclei were visualised by staining with DAPI (c, f and i). (**B**) U2OS cells were treated with LMB for 1 h in panel (a–e). The cells were fixed and stained with anti-p62 antibody (a) and anti-ARIP4 antibody (b) and visualised using Alexa488-conjugated secondary antibody and Cy3-conjugated secondary antibody, respectively. Nuclei were visualised by staining with DAPI (c). A merged image (d) shows p62 (red) and ARIP4 (green). An enlarged image (e) of the yellow-boxed area in (d) shows ARIP4 (green) and p62 (red). (**C**) The interaction between ARIP4 and p62 was visualised by *in situ* PLA assay as small, distinct spots (a and d). In LMB-treated U2OS cells, the *in situ* PLA signals were substantially increased (d). Nuclei were visualised by staining with DAPI (b and e). Merged images show PLA signal (green) and nuclei (red) (c and f). Distinct spots were not detected in cells treated with a single antibody, either anti-ARIP4 or anti-p62 alone (g and h). A representative diagram displaying the average counts of *in situ* PLA signals per nucleus (C.P.N) ±SEM. between control (Cont.) and LMB-treated cells (LMB) is shown (i). *Statistical analyses were performed with two-tailed nonparametric Mann-Whitney U-tests (n = 61 nuclei for each condition, P < 0.0001). (**D**) HEK293 cells were treated with LMB for 2 h as indicated. Nuclear extracts of HEK293 cells were subjected to immunoprecipitation using anti-ARIP4 antibody or rabbit IgG as a control. The immunoprecipitates were analysed by western blotting with the indicated antibodies. 0.5% input is shown.

To understand the interaction between p62 and ARIP4 within cells, we examined the subcellular localisation of p62 and ARIP4 in U2OS cells. We found that p62 was localised in the cytoplasm, while ARIP4 was mostly localised in the nucleus [Fig. 2A (a) and 2A (b)]. p62 shuttles between the nucleus and cytoplasm^32^; therefore, we further examined the possibility that p62 may be retained in the nucleus of U2OS cells in the presence of leptomycin B (LMB), which is a specific inhibitor of the nuclear export factor CRM1. Most p62 proteins, which were initially localised in the cytoplasm, translocated to the nucleus within 30 min after the addition of LMB [Fig. 2A (a), (d) and (g)]. These data indicate that p62 shuttles between the cytoplasm and nucleus and may therefore bind to ARIP4 in the nucleus.

We next examined colocalisation of p62 with ARIP4 in the nucleus of U2OS cells treated with LMB by a high-resolution imaging. We found that p62 formed foci in the nucleus [Fig. 2B (a)] and that ARIP4 colocalised with these foci [Fig. 2B (b))]. These data suggest that once p62 translocates to the nucleus, p62 and ARIP4 form complexes within specific nuclear compartments.

To confirm the interaction between endogenous ARIP4 and endogenous p62 in U2OS cells, we performed an *in situ* proximity ligation assay (PLA)^33^,^34^. ARIP4-p62 PLA signals were detected in the nucleus of cells treated with LMB [Fig. 2C (d–f)]. Interestingly, ARIP4-p62 PLA signals were also detected in the nuclei of non-LMB treated cells [Fig. 2C (a–c)]. Counting of the nuclear PLA signals in each condition showed that these signals were significantly increased when treated with LMB [Fig. 2C (a), (d) and (i); P < 0.0001]. As a negative control, we used a single antibody, either anti-ARIP4 or anti-p62 [Fig. 2C (g) and (h)], and confirmed the absence of any PLA signal.

To further confirm the interaction between ARIP4 and p62 in the presence of LMB, we examined whether endogenous ARIP4 could bind endogenous p62 with or without LMB treatment. We performed immunoprecipitation experiments using an anti-ARIP4 antibody and nuclear extracts of HEK293 cells, and found that p62 was co-immunoprecipitated with ARIP4 in greater quantities in nuclear extracts treated with LMB than in untreated nuclear extracts (Fig. 2D). Taken together, these data suggest that ARIP4 and p62 interact in the nucleus and that the level of this interaction is increased by treatment with LMB.

### Regions of ARIP4 responsible for interaction with p62

**Figure 3:**
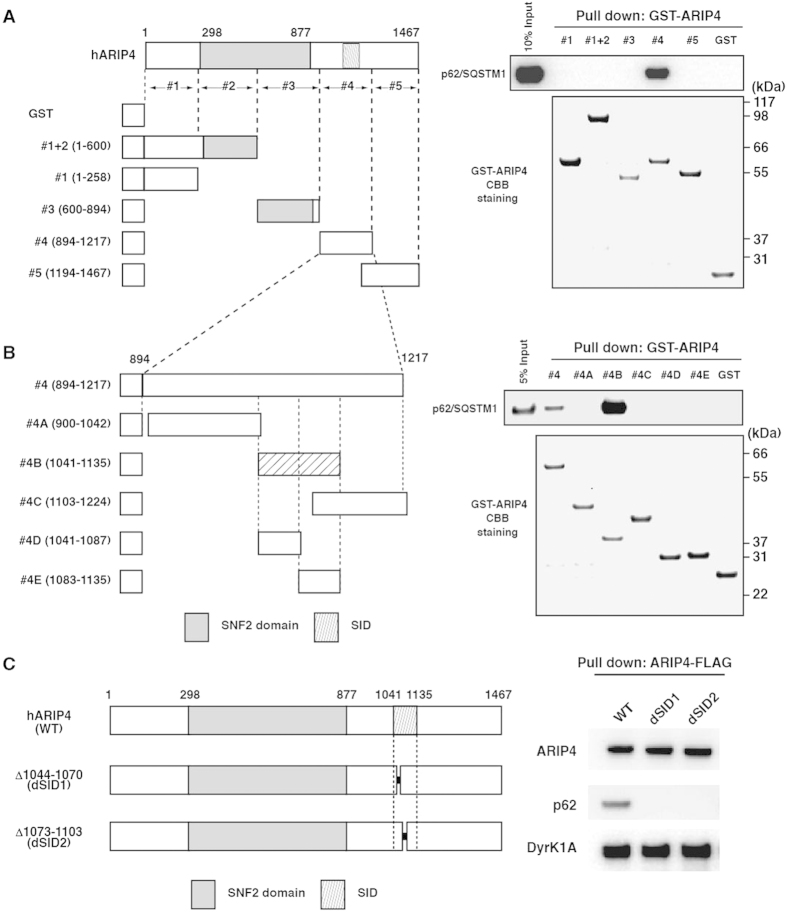
p62-binding domain of ARIP4. (**A**) Full length ARIP4 and truncated GST-fusion proteins are represented schematically. The numbers indicate the positions of amino acid residues. The SNF2 domain is represented by a shaded box. The GST-fused ARIP4 proteins were incubated with HeLa cell nuclear extracts (right-hand panel: GST-ARIP4 CBB staining of A). Glutathione Sepharose precipitates were immunoblotted with anti-p62 antibody. 10% input is shown. (**B**) Further truncated GST-ARIP4 fusion proteins investigated are also represented schematically. The numbers indicate the positions of amino acid residues. SID is represented by a hatched box. The GST-fused ARIP4 proteins were incubated with HeLa cell nuclear extracts (right-hand panel: GST-ARIP4 CBB staining of B). Glutathione Sepharose precipitates were immunoblotted with anti-p62 antibody. 5% input is shown. (**C**) p62 interacts with ARIP4 through the SID. As illustrated on the left, FLAG-tagged wild-type ARIP4 (WT) and ARIP4 mutants carrying an internal deletion of 1044–1070 a.a. (dSID1) or 1073–1103 a.a. (dSID2) were prepared. These proteins were expressed in HEK293 cells and subjected to binding assays using anti-FLAG M2 antibody. As indicated on the right, the anti-FLAG M2 agarose precipitates were immunoblotted with anti-ARIP4, anti-p62 and anti-Dyrk1A antibodies.

To determine the domains of ARIP4 that mediate binding to p62, various glutathione S-transferase (GST)-tagged fragments of ARIP4 were prepared (Fig. 3A left panel and 3B left panel) and subjected to pull-down assays from HeLa cell nuclear extracts^10^. Region #4 (894–1217) of ARIP4 bound to p62, whereas regions #1, #1+2, #3 and #5 and GST alone, did not (Fig. 3A, right upper panel). To define the binding domain within region #4, shorter GST-tagged fragments of region #4 were prepared and subjected to pull-down assays from HeLa cell nuclear extracts. Region #4B (1041–1135) interacted with p62, whereas regions #4A, #4C and GST alone, did not (Fig. 3B, right upper panel). Interestingly, although regions #4D and #4E overlapped with #4B, neither #4D nor #4E bound to p62 (Fig. 3B, right upper panel). These data suggest that the entire sequence within region #4B of ARIP4 is essential for p62 binding. We therefore named this region p62/SQSTM1 Interaction Domain (SID).

To confirm the interaction activity of SID, immunoprecipitations were performed from HEK293 cells expressing various mutants of the SID region including two internal deletion mutants, using an anti-FLAG M2 antibody (Fig. 3C, left panel). Both ARIP4 internal deletion mutants, dSID1(Δ1044–1070) and dSID2(Δ1073–1103), showed substantially reduced binding to p62 compared with the full-length wild-type ARIP4 (WT) control. However, the binding of these mutants to Dyrk1A remained unaltered (Fig. 3C, right panels), suggesting that these mutation sites are specific to p62 binding. These results show that the SID is essential for ARIP4 interaction with p62.

### Regions of p62 responsible for interaction with ARIP4

**Figure 4:**
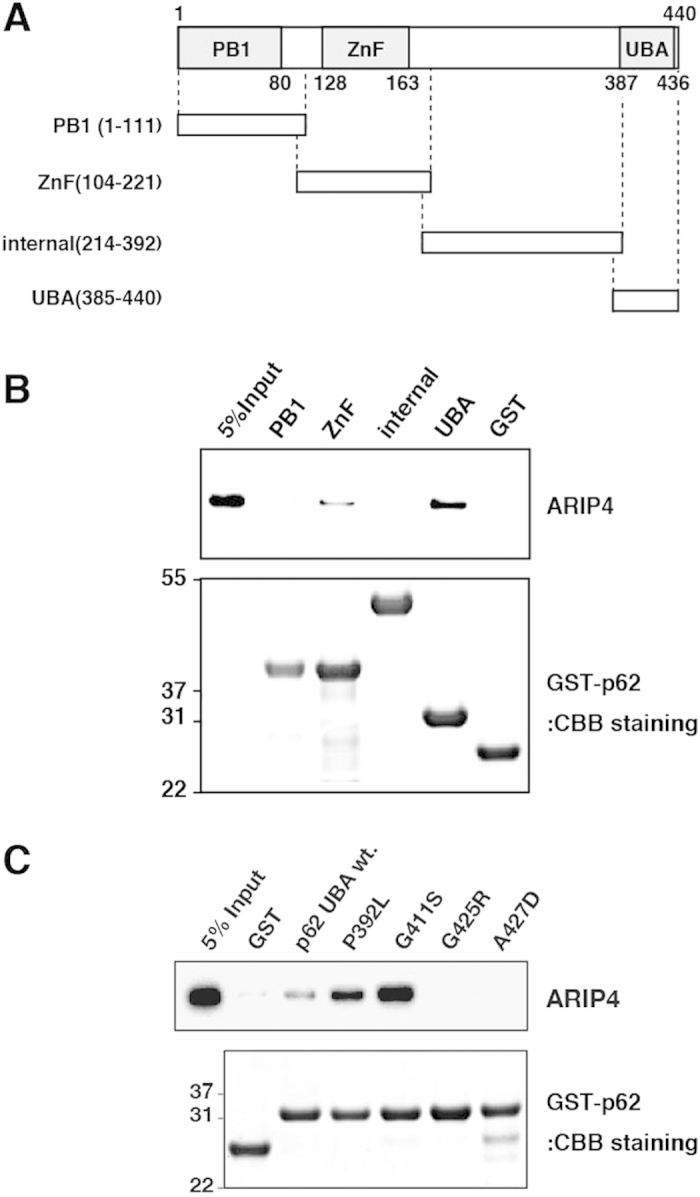
ARIP4-binding domain of p62. (**A**) The schematic depicts the truncated forms of p62 fused with GST. The numbers indicate the positions of amino acid residues. PB1, ZnF and UBA domains are represented by shaded boxes. (**B**) The GST-fused truncated forms of p62 proteins were separated by NuPAGE and stained with CBB (lower panel). The fusion proteins were incubated with recombinant ARIP4 protein. Proteins bound to Glutathione Sepharose were analysed by western blotting with anti-ARIP4 antibody (upper panel). 5% input was used as a control. (**C**) The GST-fused wild-type and human mutant UBA domains were separated by NuPAGE and stained with CBB (lower panel). The fusion proteins were incubated with recombinant ARIP4 protein. Proteins bound to Glutathione Sepharose were analysed by western blotting with anti-ARIP4 antibody (upper panel). 5% input was used as a control.

The major domain structure of p62 is illustrated in Fig. 4A. The structure includes a Phox and Bem1 (PB1) domain, a ZZ-type zinc finger (ZnF) and a UBA domain (Fig. 4A^35^,^36^). To determine the regions of p62 essential for its interaction with ARIP4, GST-tagged fragments of p62 were prepared (Fig. 4A) and subjected to pull-down assays with purified recombinant ARIP4. The UBA and the ZnF domains were observed to interact with ARIP4, whereas the PB1 domain, the internal domain and GST alone, did not. Binding between the ZnF domain and ARIP4 was substantially lower than that with the UBA domain (Fig. 4B, upper panel). These results indicate that the UBA domain of p62 is responsible for binding ARIP4.

We further examined several mutations of the p62 UBA domain that were previously identified in human patients suffering from Paget’s disease of bone (PDB)^35^,^37^. GST pull-down analysis indicated that G425R and A427D mutants failed to bind ARIP4, while P392L and G411S mutants had significantly increased binding activity (Fig.4C, upper panel). These data demonstrate that the binding characteristics between ARIP4 and the UBA domain of p62 are very similar to those reported for ubiquitin^35^,^37^.

### Ectopically expressed p62 affects the localisation of ARIP4

**Figure 5:**
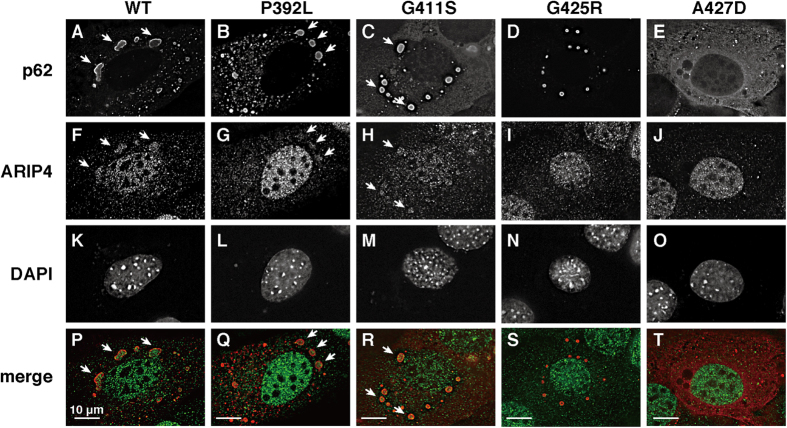
Ectopically expressed p62 forms aggresomes containing endogenous ARIP4 in the cytoplasm of p62 KO MEF cells. p62 KO MEF cells were transfected with either FLAG-p62 or FLAG-p62 mutants (P392L, G411S, G425R or A427D). After 48 h, the cells were fixed and subjected to an immunofluorescence assay using anti-ARIP4 (green) and anti-FLAG antibodies (red). The cell nuclei were stained with DAPI. Aggresomes are indicated with arrows in the images.

Mutations in the p62 UBA domain found in PDB patients showed variable binding to ARIP4 *in vitro* (Fig. 4C); therefore, we tested whether the p62 mutants affected ARIP4 localisation. We used p62 knockout (KO) MEF cells to prevent any unexpected effect of endogenous p62 on the localisation of ARIP4. Ectopic expression of wild-type p62 formed aggresomes in the cytoplasm (Fig. 5A) and these aggresomes were relatively larger than the endogenous aggresomes in the cytoplasm [compare Fig. 2A (a) with Fig. 5A]. These subcellular localisation profiles of ectopically expressed p62 are consistent with previous reports^32^,^38^. Interestingly, the aggresomes were found to contain endogenous ARIP4 (indicated by arrows in Fig. 5F,P). Furthermore, P392L and G411S mutants also formed aggresomes in the cytoplasm, which also contained ARIP4 (indicated by arrows in Fig. 5Q,R). Although the G425R mutant similarly formed aggresomes in the cytoplasm, these did not contain endogenous ARIP4 (Fig. 5S). For the A427D mutant, ARIP4 was localised in the cytoplasm but no aggresomes were observed, and ARIP4 was also localised in the nucleus (Fig. 5T). These localisation patterns appear to reflect the relative binding affinity between ARIP4 and wild-type and mutant p62.

### The SID of ARIP4 and ubiquitin interact with a similar region within the p62 UBA domain

**Figure 6:**
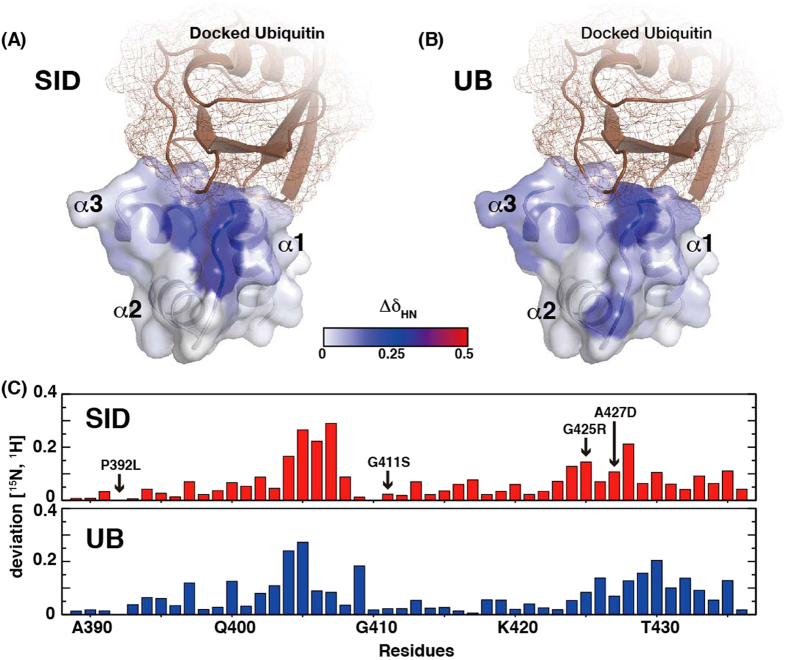
Binding interface between p62 UBA and ARIP4 SID. The UBA domain structure of p62 (PDB: 3B0F) is represented schematically in grey. The protein in brown represents a bound ubiquitin with a modelled from the complex structure of the UBA domain of Dsk2 and ubiquitin (PDB: 1WR1). (**A**) Chemical shift deviation from monomeric p62 UBA alone to SID-bound p62 UBA. (**B**) Chemical shift deviation from monomeric p62 UBA alone to ubiquitin-bound p62 UBA. Combined chemical shift deviations (^1^H and ^15^N) were plotted using the colour scheme represented (see figure centre). (**C**) Combined chemical shift deviation data were plotted on a bar graph. G410 was not observed in the UBA domain of p62 during the SID titration experiment. Accordingly, it is not included in the bar graph. Deviations in binding between the UBA domain of p62 and ubiquitin were taken from a previous report^39^ and recalculated. Combined chemical shift changes were calculated using the equation (δH^2^/2 + δN^2^/50)^1/2^, where δH and δN represent the chemical shift changes in the ^1^H and ^15^N dimensions. The residues highlighted by arrows represent the human PDB mutation sites that were tested in the pull-down analysis (Fig. 4C).

The p62 UBA domain has been reported to function primarily as a ubiquitin interaction site^39^. To assess whether the interaction between the p62 UBA domain (391–436) and ARIP4 SID (1044–1135) has a similar interaction mode to that between p62 and ubiquitin, we employed NMR titration experiments to observe the chemical shift changes in the p62 UBA domain on ARIP4 SID binding. Purified ARIP4 SID was titrated against ^15^N-p62 UBA. Specificity and mode of binding between ARIP4 SID and UBA were compared with those induced upon ubiquitin binding^39^. The NMR titration experiments showed the characteristic chemical shift deviation pattern of ^15^N-p62 UBA (Fig. 6A and upper panel of 6C). This result was similar to that for the binding between ubiquitin and the p62 UBA domain (Fig. 6B and lower panel of 6C)^39^, indicating that ARIP4 SID binds directly to the p62 UBA domain. This also implies that the ARIP4 SID employs a similar interaction mode with p62 as that between the p62 UBA domain and ubiquitin.

### Effect of p62 on ARIP4-mediated transcriptional repression

**Figure 7:**
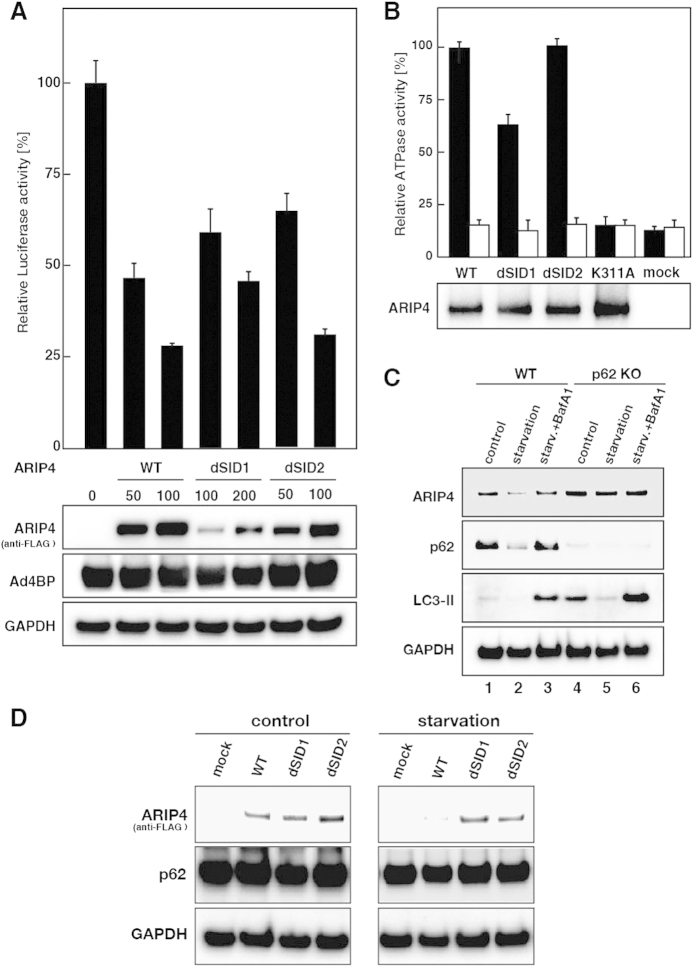
Effects of p62 on ARIP4 function. (**A**) Interaction between p62 and ARIP4 governs Ad4BP-mediated repression activity of the target promoter. HEK293 cells were transiently transfected with the luciferase reporter gene (*StAR*-Luc), Ad4BP/SF-1, and the indicated amounts (ng) of ARIP4 wild-type (WT) or ARIP4 mutants carrying an internal deletion (dSID1 or dSID2). The relative luciferase activity is shown: the amount of Ad4BP/SF-1 activation without ARIP4 expression was set at 100%. The lower panels represent ARIP4 wild-type and mutant protein levels, determined using an anti-FLAG M2 antibody. The control for the efficiency of transfection (Ad4BP/SF-1) and the loading control (GAPDH) are shown in separate panels. (**B**) The ATPase activity of ARIP4 does not modulate the p62-binding domain, SID. FLAG-tagged ARIP4 was expressed in HEK293 cells and purified with anti-FLAG M2 agarose as ARIP4 complexes. Either wild-type, dSID1, dSID2 or the ATPase mutant (K311A) of ARIP4 (100 ng) was incubated with dsDNA (1 μg). The relative ATPase activity is shown: the ATPase activity of ARIP4 WT was set at 100%, and the data are represented as the mean ± SD (upper panels; n = 3). ARIP4 WT and mutant protein levels were determined using western blot analysis with anti-ARIP4 antibody (lower panels). (**C**) Protein levels of ARIP4 and p62 decreased under nutrient starvation conditions. Wild-type and p62 KO MEF cells were cultured in starvation medium lacking amino acids and serum for 2 h, with or without the autophagy inhibitor, BafA1. Cell lysates were analysed by immunoblotting using the indicated antibodies. (**D**) Protein levels of ectopically expressed ARIP4 decreased under nutrient starvation conditions in U2OS cells. FLAG-tagged ARIP4 (WT) and their SID deletion mutants (dSID1 and dSID2) were expressed in U2OS cells. These cells were cultured in starvation medium lacking amino acids and serum for 6 h. Cell lysates were analysed by immunoblotting using the indicated antibodies.

To elucidate how p62 affects the function of ARIP4, we first examined the effect of p62 on ARIP4-mediated transcriptional repression. We have previously shown that ARIP4 acts as a coregulator to suppress transcription through its interaction with Ad4BP/SF-1. To test the effect of the direct interaction between ARIP4 and p62, we used a reporter assay to examine whether ectopically expressed ARIP4 SID deletion mutants suppressed the transcription mediated by Ad4BP/SF-1^10^. In the presence of Ad4BP/SF-1, transcription of the reporter gene was dramatically increased (Fig. 7A). Conversely, transcriptional activity was substantially reduced in the presence of WT ARIP4. Moreover, this reduction in transcription was ARIP4 protein dose-dependent. Remarkably, transcription was also considerably reduced in the presence of the SID deletion mutants, ARIP4 dSID1 and dSID2. These reductions were also protein dose-dependent (Fig. 7A). These results indicate that the ARIP4 SID is not essential for the suppression of Ad4BP/SF-1 transcriptional activity. Therefore, direct interaction with p62 is not required for ARIP4-mediated suppression of transcription.

### ATPase activity of ARIP4 does not depend on ARIP4 SID

ARIP4 contains an SNF2 domain with an ATPase motif^40^,^41^, and previous reports have shown that recombinant ARIP4 exhibits ATPase activity^9^,^10^,^42^. We examined whether the ATPase activity of ARIP4 is modulated through its interaction with p62. FLAG-tagged ARIP4 (ARIP4-FLAG) and its SID deletion mutants (ARIP4-FLAG dSID1 and ARIP4-FLAG dSID2) were purified with an anti-FLAG M2 antibody from HEK293 cells ectopically expressing FLAG-tagged ARIP4 or its mutant proteins. ARIP4-FLAG exhibited ATPase activity in the presence of dsDNA (Fig. 7B). ARIP4-FLAG dSID1 and ARIP4-FLAG dSID2 also exhibited ATPase activity. As indicated previously^42^, K311A, an ARIP4 ATPase mutant, as well as a mock purification fraction, showed no ATPase activity (Fig. 7B). These data indicate that ARIP4 SID is not required for the ATPase activity of ARIP4.

### ARIP4 protein levels are regulated by p62

Our results show that ARIP4 interacts with the UBA domain of p62 through SID (Fig. 4) and that this interaction is similar to the binding between the UBA domain and ubiquitin (Fig. 6). p62 is a well-known receptor for ubiquitinated protein in the autophagy degradation system; therefore, we hypothesised that p62 regulates ARIP4 protein levels through the autophagy pathway. To test this possibility, we first determined ARIP4 protein levels in MEF cells, in which autophagy was induced by nutrient starvation. After 2 h of nutrient starvation, ARIP4 protein levels were apparently decreased in wild-type MEF cells. Interestingly, the nutrient starvation-induced decrease in ARIP4 protein levels was not observed in p62 KO MEF cells (Fig. 7C). This indicates that ARIP4 protein levels decrease in a p62-dependent manner. To confirm that the decrease in ARIP4 protein levels was due to autophagic degradation, cells were treated with Bafilomycin A1 (BafA1), an autophagy inhibitor (see Fig. 7C, lanes 3 and 6)^28^. ARIP4 protein levels in starved wild-type MEF cells treated with BafA1 recovered to the levels of non-starvation conditions. In contrast, in p62 KO MEF cells, ARIP4 protein levels were not affected by treatment with BafA1. Furthermore, we examined whether the protein level of ARIP4 is modulated through its interaction with p62. FLAG-tagged ARIP4 (ARIP4-FLAG WT) and its SID deletion mutants (ARIP4-FLAG dSID1 and ARIP4-FLAG dSID2) were ectopically expressed in U2OS cells. After 6 h of nutrient starvation, ARIP4-FLAG WT protein levels were apparently decreased in U2OS cells. Interestingly, the nutrient starvation-induced decrease in protein levels was not observed for ARIP4-FLAG dSID1 or ARIP4-FLAG dSID2 (Fig. 7D). Taken together, these data strongly suggest that interaction between ARIP4 and p62 plays an important role in the regulation of ARIP4 protein levels under nutrient starvation conditions.

## Discussion

In the present study, we purified proteins that interact with ARIP4 and identified p62 as a major ARIP4 binding partner. Our results show that the UBA domain of p62 interacts with a novel domain in ARIP4, named SID, which possesses binding properties similar to ubiquitin. Furthermore, ARIP4 protein levels were regulated by p62 through the autophagy pathway under nutrient starvation conditions.

We showed that the primary localisation of ARIP4 and p62 was the nucleus and cytoplasm, respectively. Interestingly, LMB treatment induced substantial nuclear translocation of p62, which subsequently formed foci with ARIP4 (Fig. 2A,B). This observation is consistent with a previous report showing that p62 possesses two nuclear localisation signals (NLS) and a nuclear export signal (NES). Furthermore, p62 can be actively transported at a high rate between the cytoplasm and nucleus and can form foci on Promyelocytic Leukaemia (PML) bodies in the nucleus^32^. Our *in situ* PLA analysis showed that most interactions between ARIP4 and p62 occur in the nucleus with only a few signals observed within the cytoplasm [Fig. 2C (a–c)].

Strikingly, LMB treatment promoted the nuclear interaction between ARIP4 and p62 [Fig. 2C (d–f)]. Hocking *et al.* demonstrated that endogenous p62 is localised within the nuclei of osteoclasts and their mononuclear precursors. When osteoclasts were starved to induce autophagy, p62 and its interacting factor ALFY relocated to the cytoplasm, where they formed large aggregates^43^. This suggests that the interaction between p62 and ARIP4 is triggered by nuclear-cytoplasmic translocation of p62. Moreover, under normal conditions, a minor fraction of p62 interacts with ARIP4. It may be that p62 acts as a transporter to bring ARIP4 to various locations such as the PML body, target genes or cytoplasm.

The chemical shift perturbation pattern caused on ^15^N-p62 UBA by ARIP4 SID is similar to that caused on the UBA domain by ubiquitin (Fig. 6C). This suggests that the SID may possess a ubiquitin-like (UBL) structure. UBL domains are found in many proteins and are divided into two classes: Class 1 proteins contain a UBL domain, such as SUMO, NEDD8, ISG15, FUB1 or Urm1^44^,^45^. These proteins do not share any sequence homology, but rather a common structure including the ubiquitin fold and a C-terminal glycine residue. Moreover, they are conjugated to the lysine residue of their target proteins as a post-translational modification. In contrast, Class 2 domains cannot be conjugated to their target proteins but perform other functions via their UBL domain, such as acting as a receptor for the proteasome (hHR23a/b, hPLIC-1/2 and Ddi1)^46^, members of the ubiquitin-specific protease family (USPs)^47^, and ubiquitin E3 ligase (Parkin)^48^. The SID is an internal domain and, accordingly, it is unlikely to be conjugated to other proteins but rather dedicated for protein-protein interactions (Class 2). Furthermore, the amino acid sequence of SID is distinct from ubiquitin or other UBL domains, and sequence homology between these proteins is only 20% or less. Although the overall sequence homology between ubiquitin and the SID is very low, the region surrounding Ile44 of ubiquitin is relatively conserved within SID (1087–1102 a.a.). This supports the original hypothesis of SID sharing sequence similarities with ubiquitin, at least in the central hydrophobic core region of the ubiquitin fold^49^.

Taken together, we identified the SID as a novel UBA domain-binding region that may possess a UBL structure. This suggests that there is an unidentified intrinsic UBA-binding domain with structural homology but not amino acid sequence homology with ubiquitin, and that this domain surface resembles a region on ubiquitin.

We showed that ARIP4 was rapidly degraded after 2 h of nutritional starvation and this degradation took place through the selective autophagy pathway (Fig. 7C). Autophagy is an important catabolic process that degrades cytoplasmic components within lysosomes, thereby regulating homeostasis. It is also involved in the nutrient starvation response and various other cellular stress responses^50^,^51^,^52^. Although autophagy has long been considered an essential but non-selective degradation pathway, accumulating evidence has highlighted the importance of autophagy for selective elimination of unnecessary or unwanted components^53^. The Nrf2-Keap1 system is one of the main cellular defence mechanisms against oxidative stresses. The transcription factor, Nrf2, is constitutively degraded through the ubiquitin-proteasome pathway via its binding partner Keap1. With exposure to oxidative stress, p62 interacts with Keap1 and eliminates it through its selective autophagy pathway. As a result, p62 acts as a stress sensor to regulate the transcriptional activity of Nrf2^54^.

We have previously shown that ARIP4 binds to the nuclear receptor Ad4BP/SF-1 and modulates the SUMO-mediated fine-tuning of Ad4BP/SF-1 transcriptional activity^10^. Interestingly, our chromatin immunoprecipitation sequencing revealed that Ad4BP/SF-1 regulates nearly all genes in the glycolytic pathway^55^. Considering our finding that ARIP4 acts as a repressor of Ad4BP/SF-1, these data suggest that ARIP4 directly suppresses Ad4BP/SF-1 transcriptional activity towards sets of glycolytic pathway genes, under normal conditions. Once cells are starved of nutrition, ARIP4 may be promptly eliminated from the Ad4BP/SF-1 target promoter, translocated by p62 from the nucleus to the cytoplasm and degraded through the autophagy pathway. As a result, ARIP4 contributes to the induction of glucose metabolism gene transcription, as a defence mechanism against nutrient starvation. Hence, p62 acts as a stress sensor to contribute to a rapid increase of Ad4BP/SF-1 transcriptional activity in the glycolytic pathway. The interaction between ARIP4 and p62 therefore plays an important role in an early stage of transcriptional regulation during nutrient starvation.

## Methods

### Cell culture

HeLa S3 (ATCC CCL-2.2), HEK293 (ATCC-CRL-1573) and U2OS cells (ATCC HTB-96) were obtained from the American Type Culture Collection. MEF cells (wild-type) and p62 KO MEF cells (p62^−/−^ cells) were kindly provided by Dr. Tetsuro Ishii^56^. The cells were grown in Dulbecco’s modified Eagle’s medium (DMEM; Sigma-Aldrich) and supplemented with 10% foetal bovine serum and 1× penicillin-streptomycin-glutamine (Wako). The cells were cultured in an atmosphere of 5% CO_2_ at 37 °C. During nutrient starvation experiments, MEF cells or U2OS cells were washed twice with phosphate-buffered saline (PBS) and incubated in Hank’s balanced salt solution (HBSS; Invitrogen) containing 1 mM MgCl_2_ and 1 mM CaCl_2_ (starvation medium).

### Purification of ARIP4-containing complexes

ARIP4-containing complexes (ARIP4-binding proteins) were purified by immunoprecipitation from nuclear extracts prepared from HeLa cells expressing human ARIP4 protein fused with C-terminal FLAG- and HA-epitope tags (ARIP4-FH), using anti-FLAG antibody-conjugated agarose. The bound proteins were eluted with 3× FLAG peptide (Sigma-Aldrich) and were further affinity-purified using anti-HA (clone 12CA5) antibody-conjugated agarose as described previously^57^,^58^. Next, density gradient sedimentation was performed on 0.4 mL of the FLAG peptide-eluted material. This was loaded onto a 4-mL glycerol gradient (10–40%) and spun at 368,000 × *g* in a Beckman SW 55 Ti rotor for 6 h. Aliquots of 200 μL were sequentially collected from the top of the gradient. The proteins in each aliquot were then immunoprecipitated with anti-HA antibody-conjugated agarose and eluted with HA peptide.

### Plasmids

The ARIP4 WT and K311A expression plasmids were prepared as previously described^10^. The FLAG-tagged ARIP4 plasmid, pcDNA3-ARIP4-FLAG, was generated by modification of the 3′ end of the ARIP4 coding sequence with the PCR primers: Fw 5′-GGAGTCCTTGTGCAGAAGGTGGTCACCACG-3′, and Rev 5′-GTGTGTCTCGAGACTAGTCTACGCGGCCGCCTTTCCCAGTGACCTCTATCACATC-3′. The FLAG peptide sequence was inserted in-frame after the NotI site. The internal deletion mutant, ARIP4-FLAG dSID1 was prepared by inserting a PCR fragment into pcDNA3-ARIP4-FLAG after digestion with EcoRI and BstPI. The PCR fragment was produced with the following primers: Fw 5′-GTGTTTAGCCAGAGTCTTTCCACCTTGGCTCTCATC-3′, Rev 5′-TGTGTGTGTGGTGACCACACTTCCTCCCAATGGGACATGCCG-3′. ARIP4-FLAG dSID2 was prepared by inserting a PCR fragment into pcDNA3-ARIP4-FLAG after digestion with BstPI and NotI. The PCR fragment was produced with the following primers: Fw 5′-ACACACACAGTGGTCACCAAAGGGACGTACATCCGTACCAG-3′, Rev 5′-GTGTGTGCGGCCGCCTTTCCCAGTGACCTCTATCAC-3′. Bacterial expression plasmids of GST-fused ARIP4 deletion mutants (#4A, #4B, #4C, #4D and #4E) were constructed by subcloning PCR products of WT ARIP4 cDNA into EcoRI and XhoI sites of pGEX-6P-1 (GE Healthcare Life Sciences). The PCR fragments were produced with the following primers: #4A, Fw 5′-ACACACGAATTCCGGAAAGAGGTGGAAAACCTACTG-3′, and Rev 5′-GTGTGTCTCGAGCTATCCTCCCAATGGGACATGCCGGGG-3′; #4B, Fw 5′-ACACACGAATTCGGAGGAAGTGTAAGCTCTGCC-3′, and Rev 5′-GTGTGTCTCGAGTCAGGCAGCCATCCGACCATCTTCAGG-3′; #4C, Fw 5′-ACACACGAATTCGGGACAAAAGGGACGTACATC-3′, and Rev 5′-GTGTGTCTCGAGCTAAGGCTCAGTTCCGAGAGCTGTCCC-3′; #4D Fw 5′-ACACACGAATTCGGAGGAAGTGTAAGCTCTGCC-3′, and Rev 5′-GTGTGTCTCGAGCTAATCTGTGGAGCTGTTGAGTCCAGG-3′; #4E Fw 5′-ACACACGAATTCAACAGCTCCACAGATGTACAG-3′, and Rev 5′-GTGTGTCTCGAGTCAGGCAGCCATCCGACCATCTTCAGG-3′.

GST-fused ARIP4 deletion mutant plasmids, pGEX-ARIP4 (#1+2, #1, #3, #4 and #5) were prepared as previously described^10^.

The expression plasmid for FLAG-p62 was prepared by inserting human p62 cDNA (from the Invitrogen MGC collection) into pcDNA3 after digestion with EcoRI and XhoI. The fragment was produced by PCR with the following primers: Fw 5′-ACACACGAATTCATGGCGTCGCTCACCGTGAAGGCC-3′, and Rev 5′-GTGTGTCTCGAGTCACAACGGCGGGGGATGCTTTGA-3′. The FLAG peptide sequence was inserted in-frame before the EcoRI site. The UBA domain mutants, FLAG-p62 P392L, G411S, G425R and A427D were produced by site-directed mutagenesis of the wild-type FLAG-p62 plasmid with the following primers: P392L, Fw 5′-CCAGAGGCTGACCTGCGGCTGATTGAGTCC-3′, Rev 5′-GGACTCAATCAGCCGCAGGTCAGCCTCTGG-3′; G411S, Fw 5′-GATGAAGGCAGCTGGCTCACCAGG-3′, Rev 5′-CCTGGTGAGCCAGCTGCCTTCATC-3′; G425R, Fw 5′-TATGACATCCGAGCGGCTCTGGACACC-3′, Rev 5′-GGTGTCCAGAGCCGCTCGGATGTCATA-3′; A427D, Fw 5′-GACATCGGAGCGGATCTGGACACC-3′, Rev 5′-GGTGTCCAGATCCGCTCCGATGTC-3′.

GST-p62 deletion mutants and UBA mutant bacterial expression plasmids were constructed by subcloning PCR products of WT or mutant p62 cDNA into EcoRI and XhoI sites of pGEX-6P-1. The PCR fragments were produced with the following primers: PB1, Fw 5′-ACACACGAATTCATGGCGTCGCTCACCGTGAAGGCC-3′, and Rev 5′-GTGTGTCTCGAGTCATGGGCGGTGGTCCCGCCGGCACTC-3′; ZnF, Fw 5′-ACACACGAATTCGAGTGCCGGCGGGACCACCGCCCA-3′, and Rev 5′-GTGTGTCTCGAGTCACGTGGGGCCAGGGCGGGCCTCCCC-3′; Internal, Fw 5′-ACACACGAATTCGGGGAGGCCCGCCCTGGCCCCACG-3′, and Rev 5′-GTGTGTCTCGAGTCACGGGTCAGCCTCTGGCGGGAGATG-3′; UBA, Fw 5′-ACACACGAATTCCATCTCCCGCCAGAGGCTGACCCG-3′ and Rev 5′-GTGTGTCTCGAGTCACAACGGCGGGGGATGCTTTGA-3′.

### GST pull-down assays

Various regions of ARIP4 were expressed as GST fusion proteins in *E. coli*. The *E. coli* cells were then sonicated in a buffer containing 20 mM Tris-HCl (pH 8.0), 100 mM KCl, 10% glycerol, 5 mM MgCl_2_ and 0.1% Tween 20. Following centrifugation at 70,000 × *g* for 20 min, the supernatants were incubated with HeLa nuclear extracts and Glutathione Sepharose at 4 °C for 2 h. The beads were recovered by centrifugation, washed five times with the above buffer, and the proteins bound to the beads were then eluted with LDS sample buffer (Life Technologies) and subjected to western blot analysis with anti-p62 antibody (Sigma-Aldrich).

### Immunofluorescence Microscopy

Cells were fixed in 4% paraformaldehyde for 15 min at room temperature. The cells were then blocked in Blocking One (Nacalai Tesque, Inc.) for 30 min at 4 °C, rinsed once with 0.1% Tween-20 in PBS (PBST), incubated with rabbit polyclonal ARIP4 antibody and mouse monoclonal p62 antibody in Can Get Signal Immunostain solution A (Toyobo) for 2 h, followed by three washes with PBST and then incubated with Alexa488-conjugated anti-rabbit IgG antibody and Cy3-conjugated anti-mouse IgG antibody (Life Technologies) for 1 h. After three washes with PBST, the cells were stained with DAPI and examined by fluorescence microscopy using an Olympus Fluoview FV1000 laser scanning confocal microscope.

### *In situ* PLA analysis

*In situ* PLA analysis^33^,^34^ was carried out in accordance with the manufacturer’s protocol (Duolink *in situ* PLA kit; Sigma-Aldrich). U2OS cells in glass bottom dishes were fixed in 4% paraformaldehyde. The cells were then blocked in Blocking One for 30 min at 4 °C, incubated with primary antibodies (mouse anti-p62/SQSMT1 antibody [1:1000] and rabbit anti-ARIP4 antibody [1:500]) in antibody diluent solution overnight at 4 °C. The cells were then washed three times for 3 min with PBST and incubated with PLA probes, anti-mouse PLUS and anti-rabbit MINUS (Duolink *in situ* PLA kit; Sigma-Aldrich), mixed and diluted in 1× antibody diluent, for 2 h at 37 °C. The cells were then washed with PBST for 3 min with gentle agitation, incubated with Duolink Ligase in ligation solution at a 1:40 dilution for 15  min at 37 °C, washed with PBST, and incubated with amplification solution containing DNA polymerase for 90 min at 37 °C. The cells were then washed three times, and incubated with 6.25 nM fluorescence-labelled probe (Detection Kit 563; Sigma-Aldrich) for 30 min at 37 °C. After extensive washing in decreasing concentrations of SSC buffers (2×, 1×, 0.2×, 0.02×; 2 minutes each), the cells were mounted using Duolink Mounting Medium, and photographed with a DeltaVision RT system (Applied Precision). Statistical analyses were performed using the nonparametric, unpaired Mann-Whitney U-test using Graphpad Prism software.

### Expression and purification of ARIP4 SID for NMR titration assay against p62 UBA

ARIP4 SID (1044–1135) was expressed as an N-terminal GST-tagged SUMO1 fusion protein in *E. coli*. Initial purification was performed on a Glutathione Sepharose 4FF column (GE Healthcare Life Sciences). After the removal of the GST-SUMO1 tag by a SUMO-specific protease, ARIP4 SID was purified via cation exchange and size-exclusion chromatography. The p62 UBA domain (391–438) was purified as described previously^39^. Both samples were subsequently transferred to a buffer containing 20 mM sodium phosphate (pH 6.8), 5 mM potassium chloride and 1 mM EDTA.

### NMR titration of ARIP4 SID against the p62 UBA domain

Non-labelled ARIP4 SID (1044–1135) was titrated against 50 μM of the ^15^N-labeled human p62 UBA domain to twice the UBA molarity. The equation for combined chemical shift deviation, δHN = (δH^2^/2 + δN^2^/50)^1/2^, was used to calculate deviations at each peak, where δH and δN represent proton and nitrogen minimal deviation from the assigned free-form monomeric UBA peaks to the unassigned SID-bound peaks, respectively (at double the molarity of SID to UBA).

### Reporter gene assays

Cells were seeded at a density of 6 × 10^4^ cells per well (24-well plate), cultured for 24 h and transfected with plasmids using Lipofectamine 2000 (Invitrogen) according to the manufacturer’s protocol. The total amount of transfected plasmid was adjusted to 550 ng with vector plasmids. pRL-SV40 (*Renilla* Luciferase control reporter vector; Promega) was used as an internal control to normalise transfection efficiency. The cells were harvested 36 h post transfection and subsequently assayed for luciferase activity. The luciferase assays were performed using a dual-luciferase reporter assay system (Promega).

### ATPase assays

Cells were seeded at a density of 2 × 10^6^ cells per well (100-mm dish), cultured for 24 h and transfected with plasmids using Lipofectamine 2000 according to the manufacturer’s protocol. The total amount of transfected plasmid was adjusted to 20 μg with vector plasmids. Recombinant FLAG-tagged ARIP4 proteins were produced in HEK293 cells and purified as described previously^10^. The ATPase activity of ARIP4 was determined using Biomol Green reagent (Biomol Research Laboratories) according to the manufacturer’s protocol. Purified FLAG-tagged ARIP4 was incubated in a 50 μL reaction containing 20 mM Tris-HCl (pH 8.0), 50 mM KCl, 5 mM MgCl_2_ and 200 μM ATP in the presence of dsDNA. pBlueScript SK(+) was used as the dsDNA.

## Additional Information

**How to cite this article**: Tsuchiya, M. *et al.* Selective autophagic receptor p62 regulates the abundance of transcriptional coregulator ARIP4 during nutrient starvation. *Sci. Rep.* **5**, 14498; doi: 10.1038/srep14498 (2015).
